# Supplementary material for: Structural and molecular determinants of Candida glabrata metacaspase maturation and activation by calcium
Source: Commun Biol. 2022 Oct 31;5:1158. doi: 10.1038/s42003-022-04091-4 (PMC9622860; doi:10.1038/s42003-022-04091-4)
Supplement: Supplementary file 2 — Supplementary Material [file 42003_2022_4091_MOESM2_ESM.pdf]

Supplementary information

Conchou L. et al. 2022

Supplementary Tables

Primers	Sequence
**External primers**	
*Sal*I	5'- C GGC GTC GAC ATC CCG GGC AGC GGT AAC-3'
*Sac*I	5'-CG GAT GAG CTC CAT AAT AAA CTG CAG G-3'
**Internal primers**	
C238A	
sense	5' - CTGACCGCGCTGTTTGATAGCGCGCACAGCGGTACCGTTCTGGA - 3'
antisense	5' - TCCAGAACGGTACCGCTGTGCGCGCTATCAAACAGCGCGGTCAG - 3'
C238A/H182A	
sense	5' - TGTTCTTTCACTACAGCGGTGCGGGCGGTCAAACCAAAGACCTG - 3'
antisense	5' - CAGGTCTTTGGTTTGACCGCCCGCACCGCTGTAGTGAAAGAACA - 3'
R54A	
sense	5'-GGGCGAGAACGCGGGCCAATACCAGGG-3'
antisense	5'-CCCTGGTATTGGCCCGCGTTCTCGCCC-3'
K263A	
sense	5'-GCCGAACATGTGGGCGGATGTTGGTAGCG-3'
antisense	5'-CGCTACCAACATCCGCCCACATGTTCGGC-3'
K263D	
sense	5'-GCCGAACATGTGGGATGATGTTGGTAGCG-3'
antisense	5'-CGCTACCAACATCATCCCACATGTTCGGC -3'
K263F	
sense	5'-GCCGAACATGTGGTTTGATGTTGGTAGCG -3'
antisense	5'-CGCTACCAACATCAAACCACATGTTCGGC -3'
R307A	
sense	5'-GGACCGTGAACGTGTTGCGCAAATCAAGTTTAGCC -3'
antisense	5'- GGCTAAACTTGATTTGCGCAACACGTTCACGGTCC -3'

Supplementary Table 1. List of oligonucleotide primers. All primers were custom synthesized at Sigma-Aldrich, India

Constructs	^{His}*Cg*MCA-I	*Cg*MCA-I^{His}
**Expression vector**	pET15b	pET52
***Cg*MCA-I gene length (bp)**	1179	
***Cg*MCA-I amino acids number**	392	
***Cg*MCA-I size (kDa)**	45	
**Recombinant *Cg*MCA-I amino acids number**	432	443
**Recombinant *Cg*MCA-I size (bp)**	47.9	49.7

Supplementary Table 2. ^{His}*Cg*MCA-I and *Cg*MCA-I^{His} constructs characteristics.

Tampon	Composition
Lysis buffer	
∅	HEPES 10 mM pH 7.6, NaCl 150 mM
Ion	HEPES 10 mM pH 7.6, NaCl 150 mM, $MnCl_2$ or $MgCl_2$ or $ZnCl_2$ 10 mM
Washing buffer	
∅	HEPES 10 mM pH 7.6, NaCl 150 mM, imidazole 30 mM
Ion	HEPES 10 mM pH 7.6, NaCl 150 mM, imidazole 30 mM, $MnCl_2$ or $MgCl_2$ or $ZnCl_2$ 10 mM
Elution buffer	
∅	HEPES 10 mM pH 7.6, NaCl 150 mM, imidazole 500 mM
Ion	HEPES 10 mM pH 7.6, NaCl 150 mM, imidazole 500 mM, $MnCl_2$ or $MgCl_2$ or $ZnCl_2$

Supplementary Table 3. List of buffers for maturation assays in the presence of different divalent cations using Biosprint 96. All buffers were freshly prepared prior to experiments.

Buffer	Buffer composition
Lysis buffer	
∅	10 mM HEPES pH 7.6, 150 mM NaCl
Ca	10 mM HEPES pH 7.6, 150 mM NaCl, 10 mM $CaCl_2$
EGTA	10 mM HEPES pH 7.6, 150 mM NaCl, 1 mM EGTA
Washing buffer	
∅	10 mM HEPES pH 7.6, 150 mM NaCl, 30 mM imidazole
Ca	10 mM HEPES pH 7.6, 150 mM NaCl, 30 mM imidazole, 10 mM $CaCl_2$
EGTA	10 mM HEPES pH 7.6, 150 mM NaCl, 30 mM imidazole, 1 mM EGTA
Elution buffer	
∅	10 mM HEPES pH 7.6, 150 mM NaCl, 500 mM imidazole
Ca	10 mM HEPES pH 7.6, 150 mM NaCl, 500 mM imidazole, 10 mM $CaCl_2$
EGTA	10 mM HEPES pH 7.6, 150 mM NaCl, 500 mM imidazole, 1 mM EGTA

Supplementary Table 4. List of buffers for maturation assays using Biosprint 96. All buffers were freshly prepared prior to experiments.

Parameters	Value	Standard deviations	CV (%)
Day 0			
K_m	18.0 µM	12.0	64.7
k_{cat}	2.8 min^{-1}	0.7	24.9
k_{chem}	0.107 min^{-1}	0.012	10.8
Day 8			
K_m	18.2 µM	1.7	9.1
k_{cat}	15.8 min^{-1}	0.6	3.6
k_{chem}	0.1183 min^{-1}	0.0092	7.8

Supplementary Table 5. **Kinetic parameters for metacaspase at maturation days 0 and 8**. Data are obtained using the Dynafit software package using script 1 (*vide supra*).

Model	Reactions	Constants	Constant Order	Units
Cis	$E_{low} \rightarrow E_{high}$	k_1	first	day^{-1}
Trans A	$E_{low} + E_{low} \rightarrow E_{high} + E_{low}$	k_1	second	$\mu M^{-1}.day^{-1}$
Trans B	$E_{low} + E_{low} \rightarrow E_{high} + E_{high}$	k_1	second	$\mu M^{-1}.day^{-1}$
Trans C	$E_{low} + E_{low} \rightarrow E_{high} + E_{low}$<br>$E_{low} + E_{high} \rightarrow E_{high} + E_{high}$	k_1<br>k_2	second<br>second	$\mu M^{-1}.day^{-1}$
Trans D	$E_{low} + E_{low} \rightarrow E_{high} + E_{high}$<br>$E_{high} + E_{low} \rightarrow E_{high} + E_{high}$	k_1<br>k_2	second<br>second	$\mu M^{-1}.day^{-1}$
Cis-Trans	$E_{low} \rightarrow E_{high}$<br>$E_{high} + E_{low} \rightarrow E_{high} + E_{high}$	k_1<br>k_2	first<br>second	day^{-1}<br>$\mu M^{-1}.day^{-1}$

Supplementary Table 6. **Proposed models describing the conversion of E_{low} population into E_{high} population**.

Set number	1		2		3	
k_1	0.001	variable	0.001	variable	0.001-0.1	fixed[a]
k_2	1	variable	1	variable	1	variable
r_{low}	$1.25x10^{-6}$	variable	$1.25x10^{-6}$	fixed	$1.25x10^{-6}$	variable
r_{high}	$7.5x10^{-6}$	variable	$7.5x10^{-6}$	fixed	$7.5x10^{-6}$	variable

Supplementary Table 7. **Sets of initial parameters for model discrimination**.[a]: several fits are performed with fixed values of k_1: 0.001, 0.01 and 0.1 (see Supplementary Table 5 for the units).

Parameter	Value	CV (%)
k_1 (day^{-1})	0.001	n.a.
k_2 ($\mu M^{-1}.day^{-1}$)	0.1542 ± 0.0061	4.0
r_{low} ($\mu M.min^{-1}.\mu M^{-1}$)	$(1.33 \pm 0.14)x10^{-6}$	10.4
r_{high}($\mu M.min^{-1}.\mu M^{-1}$)	$(7.51 \pm 0.34)x10^{-6}$	4.5

Supplementary Table 8. **Optimized parameters for the Cis-Trans model (most probable model).**

Cg MCA-I D0											
Without incubation				1 hour incubation				24 hours incubation			
Protein (mg/mL)	Ligand (mM)	Tm (°C)	Delta Tm (°C)	Protein (mg/mL)	Ligand (mM)	Tm (°C)	Delta Tm (°C)	Protein (mg/mL)	Ligand (mM)	Tm (°C)	Delta Tm (°C)
1	0	45	-0.05	1	0	44.9	-0.05	1	0	47.2	0.05
1	0	45.1	0.05	1	0	45.1	0.05	1	0	47.1	-0.05
1	0.1	48.5	3.45	1	0.1	49.3	4.3	1	0.1	54.1	6.95
1	0.1	48.8	3.75	1	0.1	49.2	4.2	1	0.1	54	6.85
1	0.25	53.6	8.55	1	0.25	54	9	1	0.25	58.1	10.95
1	0.25	53.7	8.65	1	0.25	53.9	8.9	1	0.25	58.2	11.05
1	0.5	58.4	13.35	1	0.5	58.7	13.7	1	0.5	59.6	12.45
1	0.5	58.3	13.25	1	0.5	58.7	13.7	1	0.5	59.7	12.55
1	1	59.8	14.75	1	1	60	15	1	1	61.1	13.95
1	1	59.8	14.75	1	1	60.1	15.1	1	1	61	13.85
1	2	60.9	15.85	1	2	61.4	16.4	1	2	63.1	15.95
1	2	60.9	15.85	1	2	61.4	16.4	1	2	63.1	15.95
1	5	63.4	18.35	1	5	63.6	18.6	1	5	65	17.85
1	5	63.5	18.45	1	5	63.6	18.6	1	5	65	17.85
1	7	63.8	18.75	1	7	64.1	19.1	1	7	65.4	18.25
1	7	63.8	18.75	1	7	64.1	19.1	1	7	65.5	18.35
1	10	64.2	19.15	1	10	64.4	19.4	1	10	65.7	18.55
1	10	64.2	19.15	1	10	64.4	19.4	1	10	65.8	18.65
1	20	64.6	19.55	1	20	64.9	19.9	1	20	66.1	18.95
1	20	64.7	19.65	1	20	64.9	19.9	1	20	66.1	18.95

Supplementary Table 9. **Melting temperature (Tm) of *Cg*MCA-I, at maturation day 0, in a range of $CaCl_2$ concentration without or with incubation time**. Delta Tm are calculated from the average Tm_0 (Tm without any ligand) for all three conditions, without and with 1 hour or 24 hours of incubation with different $CaCl_2$ concentrations prior to fluorescence measurements.

Cg MCA-I D8											
Without incubation				1 hour incubation				24 hours incubation			
Protein (mg/mL)	Ligand (mM)	Tm (°C)	Delta Tm (°C)	Protein (mg/mL)	Ligand (mM)	Tm (°C)	Delta Tm (°C)	Protein (mg/mL)	Ligand (mM)	Tm (°C)	Delta Tm (°C)
1	0	48.8	0	1	0	49.2	0.1	1	0	49.2	0
1	0	48.8	0	1	0	49	-0.1	1	0	49.2	0
1	0.1	53.8	5	1	0.1	54.8	5.7	1	0.1	54	4.8
1	0.1	53.8	5	1	0.1	54.5	5.4	1	0.1	54.1	4.9
1	0.25	57.8	9	1	0.25	58.3	9.2	1	0.25	58.4	9.2
1	0.25	58.1	9.3	1	0.25	58.1	9	1	0.25	58.4	9.2
1	0.5	60.1	11.3	1	0.5	60.3	11.2	1	0.5	60.2	11
1	0.5	60.1	11.3	1	0.5	60.3	11.2	1	0.5	60.4	11.2
1	1	61.7	12.9	1	1	61.7	12.6	1	1	61.9	12.7
1	1	61.7	12.9	1	1	61.7	12.6	1	1	61.9	12.7
1	2	63.1	14.3	1	2	63.3	14.2	1	2	63.2	14
1	2	63.1	14.3	1	2	63.2	14.1	1	2	63.3	14.1
1	5	64.6	15.8	1	5	64.6	15.5	1	5	64.5	15.3
1	5	64.5	15.7	1	5	64.6	15.5	1	5	64.6	15.4
1	7	64.8	16	1	7	64.9	15.8	1	7	64.8	15.6
1	7	64.8	16	1	7	65	15.9	1	7	64.7	15.5
1	10	65	16.2	1	10	65.2	16.1	1	10	64.8	15.6
1	10	65	16.2	1	10	65.2	16.1	1	10	64.9	15.7
1	20	65.3	16.5	1	20	65.5	16.4	1	20	65.2	16
1	20	65.3	16.5	1	20	65.4	16.3	1	20	65.3	16.1

Supplementary Table 10. **Melting temperature (Tm) of *Cg*MCA-I, at maturation day 8, in a range of $CaCl_2$ concentration without or with incubation time**. Delta Tm are calculated from the average Tm_0 (Tm without any ligand) for all three conditions, without and with 1 hour or 24 hours of incubation with different $CaCl_2$ concentrations prior to fluorescence measurements.

Supplementary Figures

a

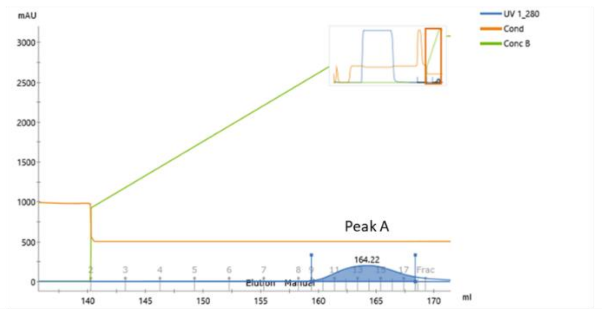

Peak	Retention (ml)	Area (ml*mAU)	Area %	Fraction(s)	Volume (ml)	Conductivity (mS/cm)
Peak A	164.216	1149	100.00	9 - 17	9.007	16.25

b

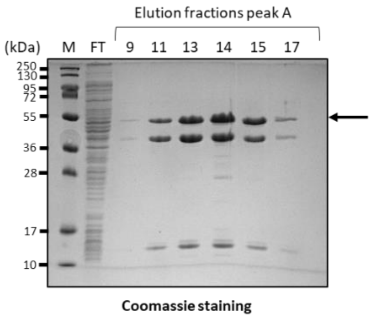

Supplementary Figure 1. Purification of *Cg*MCA-I by affinity chromatography. a. Elution chromatogram of *Cg*MCA-I using a TALON cobalt crude column. Elution was performed by a linear gradient of elution buffer (10 mM HEPES pH 7.6, 300 mM NaCl, 10 mM $CaCl_2$, 500 mM imidazole) followed by a step at 100% of elution buffer. **b**. Elution peak A fractions 11 to 17 were loaded on a 15% SDS-PAGE gel, and it can be observed that *Cg*MCA-I (48 kDa) is present in all fractions. Arrow indicates the full-length protein.

a

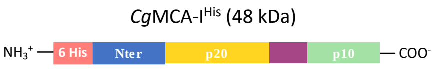

b

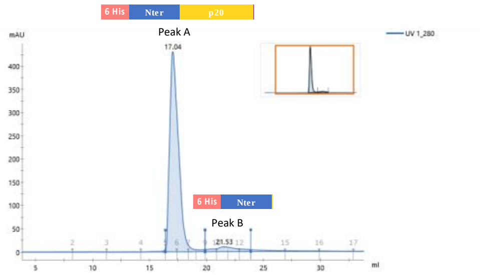

Peak	Retention (ml)	Area (ml*mAU)	Area %	Fraction(s)	Volume (ml)	Conductivity (mS/cm)
Peak A	17.042	378.6	93.61	5 - 8	3.486	30.82
Peak B	21.527	25.83	6.39	9 - 13	4.011	30.81

c

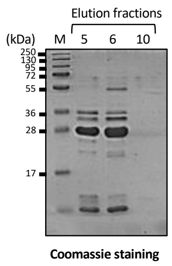

d

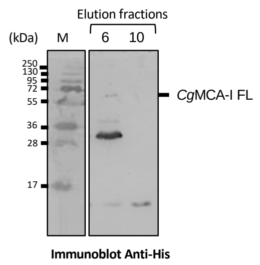

Supplementary Figure 2. Purification of *Cg*MCA-I by size exclusion chromatography. **a**. Schematic representation of *Cg*MCA-I^{His} construct with the 6xHis tag, the N-terminal part, the p20 domain, the linker and p10 domain respectively colored in *red*, *blue*, *yellow*, *purple* and *green*. **b**. Elution chromatogram of *Cg*MCA-I size exclusion chromatography using a S200 increase column. An isocratic elution was performed using an elution buffer (10 mM HEPES pH 7.6, 150 mM NaCl, 10 mM $CaCl_2$ and 1% glycerol). Parts of the protein probably corresponding to peak A and B are shown and colored as mentioned in (**a**). **c**. Elution

fractions 5, 6 from peak A and 10 from peak B were loaded on a 15% SDS-PAGE gel and fractions 5 and 6 were concentrated using an Amicon Ultra Centrifugal filter with a 30 kDa molecular weight cut-off. **d**. Immunoblot Anti-His of elution fractions 6 from peak A and 10 from peak B.

a

Monoisotopic mass (Da)	DB mass (Da)	Δppm	Peptidic sequence	Position
6470.721	6470.724	0.003	(R)GSHMYPGSGNYSYNNRPSMPPPGFNGDGQGYRQEYGNQYGGGYQQQQYQDQ YQGENR	17 - 74
9364.673	9364.685	1.2	(R)QIKFSPADVIMLSGSKDNQTSADTFADGQNIGAMSHAFISVMTRQPQQSYLSLLQ NLRNELAGKYSQKPQLSASHPIDVNLQFIM	328 - 412
23590.42	23590.43	0.2	(R)GQYQGQYQDQPEYGRPPSGMVRPPSSIQQGNGQQFQYSQMTGRRKALLIGINYI GSKNALRGCINDAHNIFNYLITTYCGYRPEDIVMLTDDQREMVKIPLKENIIRAMQWL VKDAQPNDALFFHYSGHGGQTKDLDGDEEDGMDDVIYPVDFESVGPLIDDTMHDI MVKSLPQGARLTALFDSCHSGTVLDLPYTYSTKGVIKEPNMWK	75 - 283

b

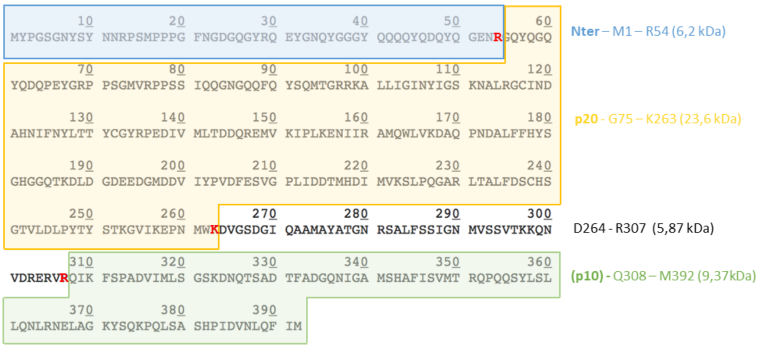

Supplementary Figure 3. Identification of cleavage sites and associated peptide sequences by mass spectrometry. **a**. The peptide sequences identified by mass spectrometry revealed three cleavage sites. Theoretical masses (DB mass) of the identified peptides were also calculated using the Findpept tool (Expasy) as well as the difference between experimental and theoretical values (Δppm). Residue numbering takes into account the 20 residues of the histidine tag. **b**. The N-terminal region, p10- and p20 subunits are indicated in *blue*, *yellow* and *green*, respectively. Cleavage sites residues are highlighted in *red*.

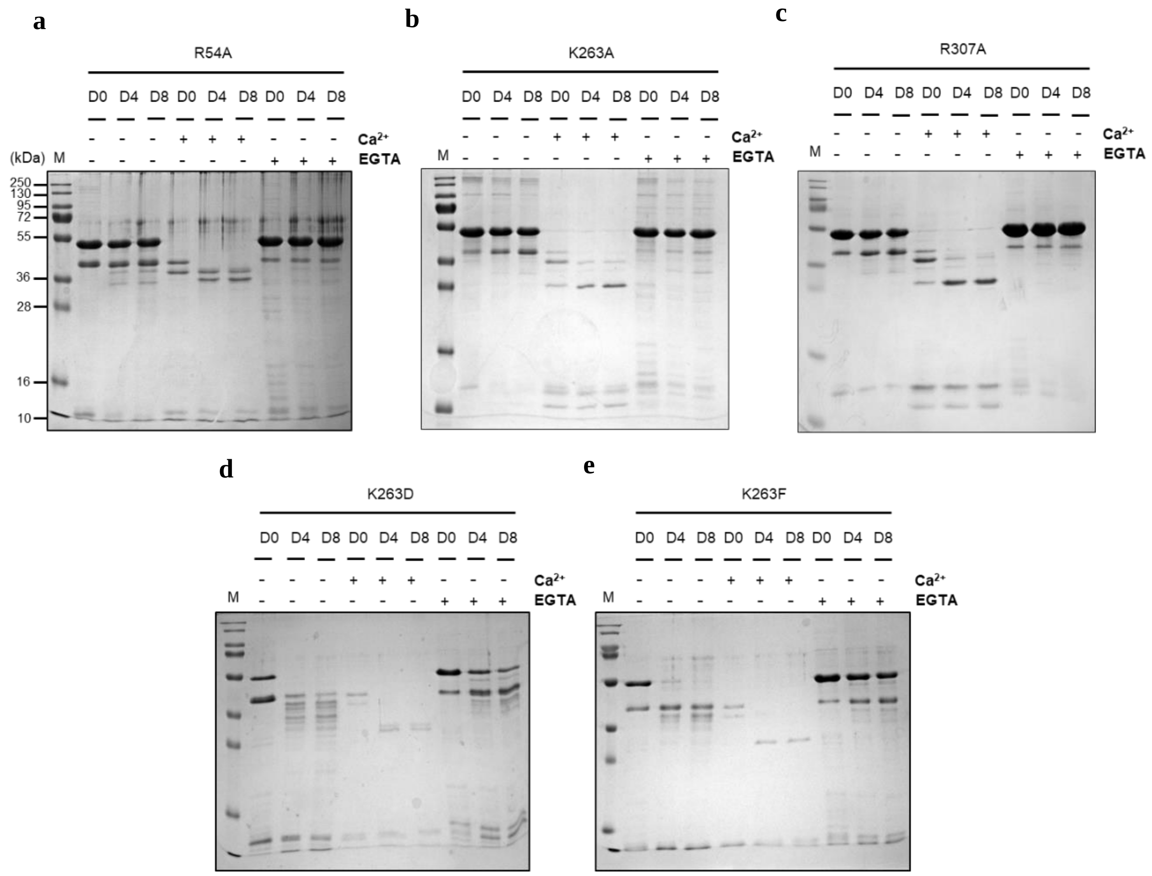

Supplementary Figure 4. Maturation of *Cg*MCA-I mutants in the presence or absence of divalent cations and EGTA. a, b, c, d, **e**. SDS-PAGE of R54A (**a**), K263A (**b**), R307A (**c**), K263D (**d**) and K263F (**e**) mutants at days 0 (D0), 4 (D4) and 8 (D8) of maturation in presence or absence of Ca^{2+} (10 mM) or EGTA (1 mM).

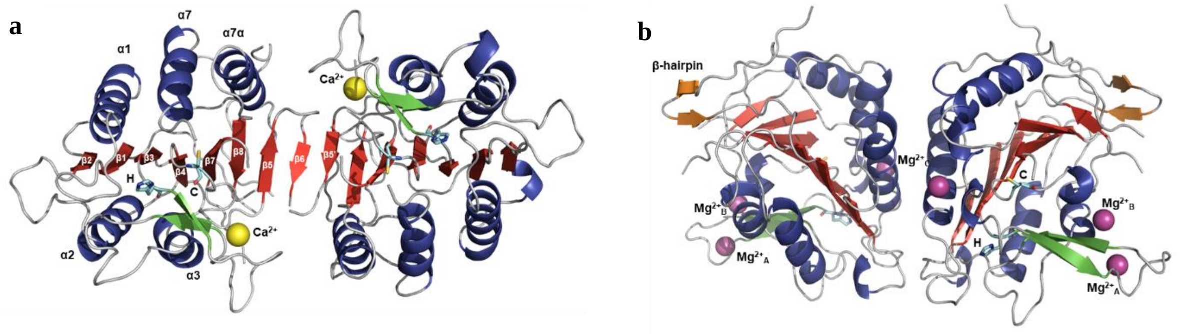

Supplementary Figure 5. Overall structural organization of *Cg*MCA-I as observed in the asymmetric unit. a, b. Three-dimensional structures of *Cg*MCA-I^{Ca} and *Cg*MCA-I^{Mg} represented with two monomers as observed in the crystal asymmetric unit respectively in complex with (**a**) Ca^{2+} (*yellow* sphere) or (**b**) Mg^{2+} (*purple* spheres). α helices (*blue*) and β strands (*red*), with the numbering of the secondary structure elements shown in *white* and *black*, respectively **(a)**. The three Mg^{2+} ions present in *Cg*MCA-I^{Mg} are named $Mg^{2+}{}_{A}$, $Mg^{2+}{}_{B}$ and $Mg^{2+}{}_{C}$ **(b).** Catalytic residues are highlighted as stick presentations by H (His^{182}) and C (Cys^{238}) and colored in *cyan*.

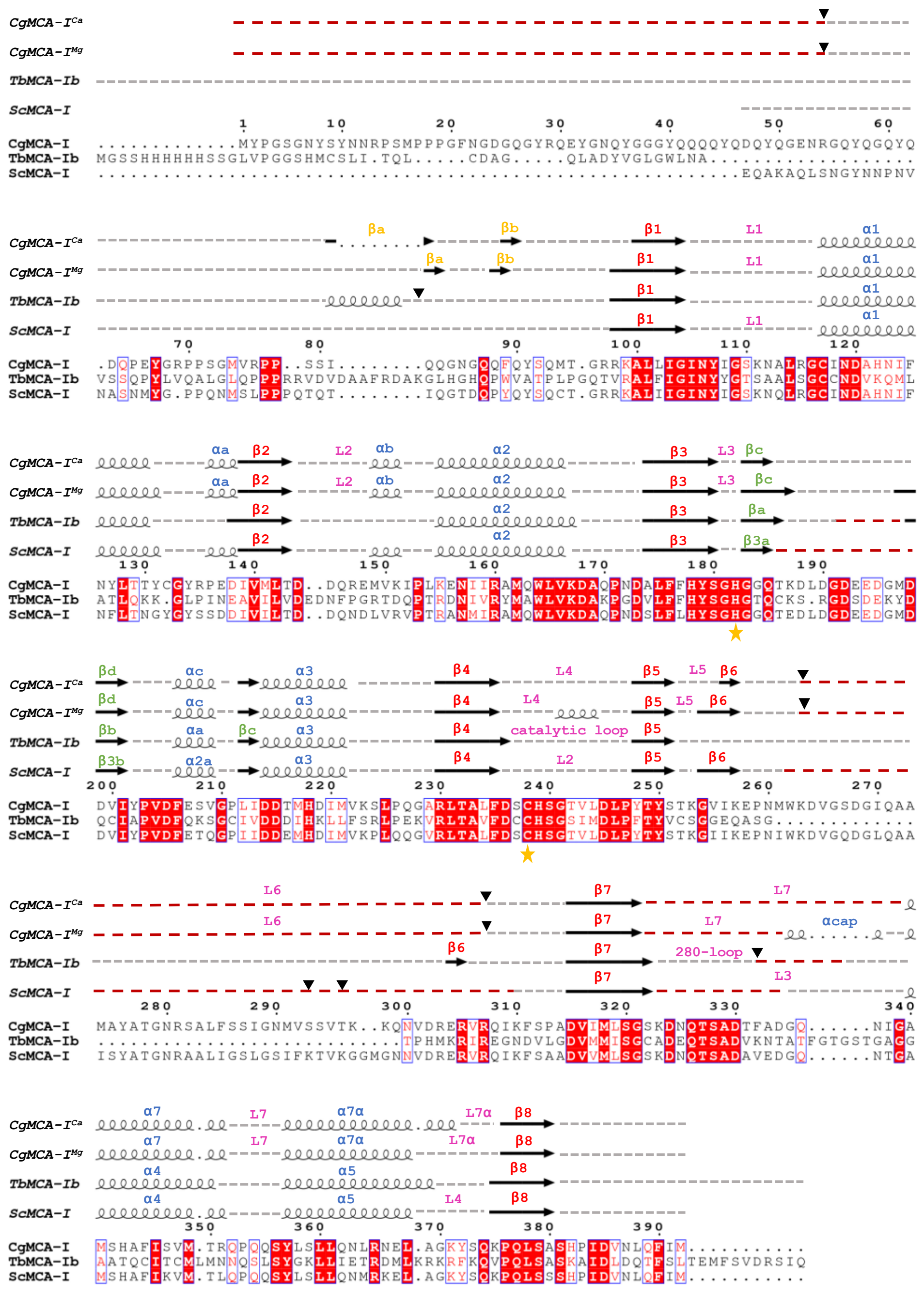

Supplementary Figure 6. Sequence and secondary structure alignment of *Cg*MCA-I^{Ca}, *Cg*MCA-I^{Mg}, *Tb*MCA-Ib and *Sc*MCA-I. Alignment of the three sequences has been performed using the web tool Clustal Omega[1], visual representation of sequence and secondary structure alignments have been performed using the web tool Espript 3.0[2]. α-Helices are numbered in *blue*, β strands are in *red*, *yellow* (βa-βb) and *green* (βc-βd), loops are in *pink* and are represented by *grey* dotted lines. The numbering of *Sc*MCA-I (PDB code: 4F6O) and *Tb*MCA-I (PDB code: 4AFP) follows the numbering used by the authors of previous publications[3,4]. The catalytic residues, His^{182} and Cys^{238}, are indicated by an *orange* star. The cleavage sites identified for each enzyme are indicated by *black* triangles.

a

b

c

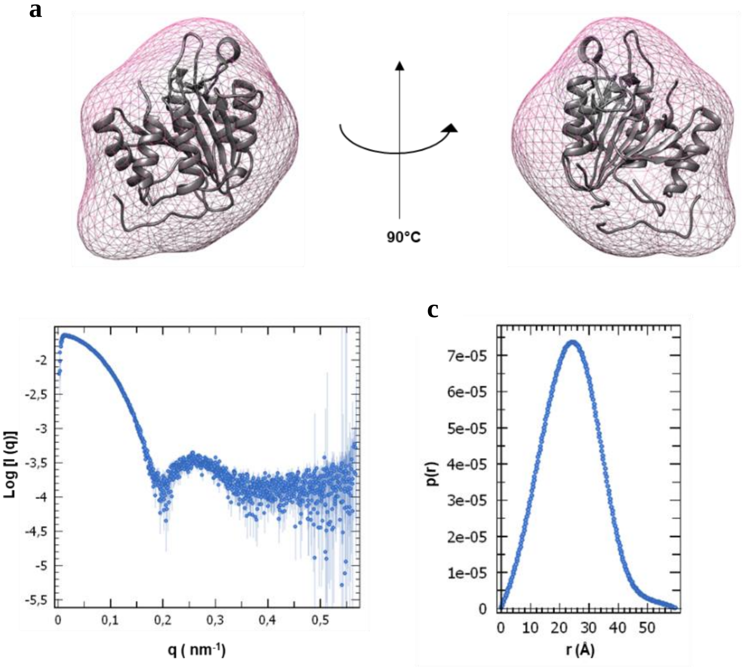

d

Protein	MW (kDa) calculated	Elution volume (mL)	Rg (Å)	MW Qp (kDa)	Dmax (nm)	Porod volume (Å³)	Oligomeric state
*Cg*MCA-I	48	2.2	18.72 ± 0.42	23	58.42	3380	Monomeric

Supplementary Figure 7. Small Angle X-ray Scattering studies of *Cg*MCA-I. a. *Ab initio* model of *Cg*MCA-I generated from the SEC-SAXS experimental data using the program DAMMIF[10]. The crystal structure of *Cg*MCA-I^{Ca} was superimposed onto the calculated (18 Å) SAXS envelope using the software CHIMERA (University of California, San Francisco) and confirmed a monomeric structure of the enzyme in solution. **b.** Log I(q) *versus* q was generated using PRIMUS[8]. **c.** P(r) *versus* r profile from the data in **(b)** was carried out using PRIMUS[8]. **d**. SAXS data for *Cg*MCA-I.

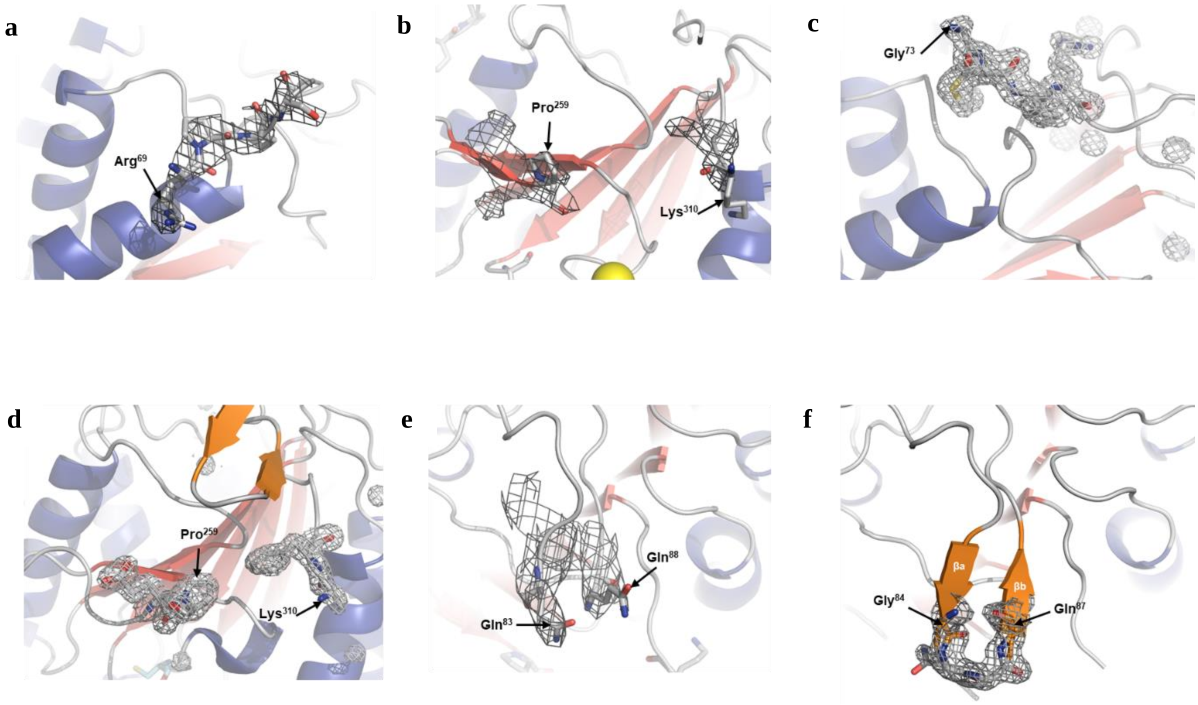

Supplementary Figure 8. Electronic density showing the extremity of missing loops as seen in *Cg*MCA-I^{Ca} and in *Cg*MCA-I^{Mg}. a. Missing amino-terminal part Met^{1}-Gly^{68} in *Cg*MCA-I^{Ca}. **b.** Missing loop Asn^{260}-Ile^{309} in *Cg*MCA-I^{Ca} **c.** Missing amino-terminal part Met^{1}-Ser^{72} in *Cg*MCA-I^{Mg}. **d.** Missing loop Asn^{260} to Ile^{309} in *Cg*MCA-I^{Mg} **e-f.** Absence of loop Gly^{84}-Gln^{87} in *Cg*MCA-I^{Ca} (**e**) as opposed to *Cg*MCA-I^{Mg} (**f**).

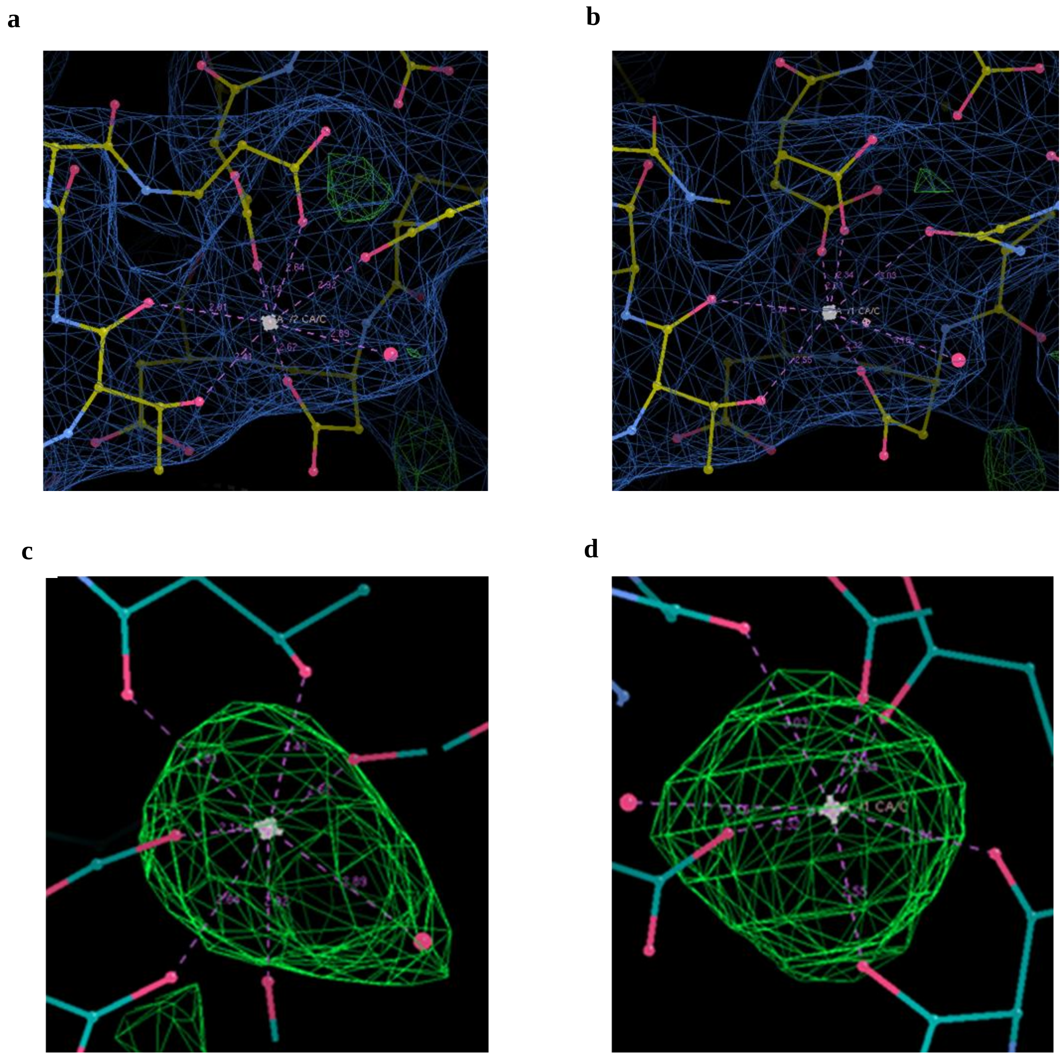

Supplementary Figure 9. First coordination sphere of Ca^{2+} in subunit A of *Cg*MCA-I^{Ca} (a) or subunit B of *Cg*MCA-I^{Ca} (b). Omit-map (5 sigma) around Ca^{2+} in subunit A (c) and B (d) of *Cg*MCA-I^{Ca}.

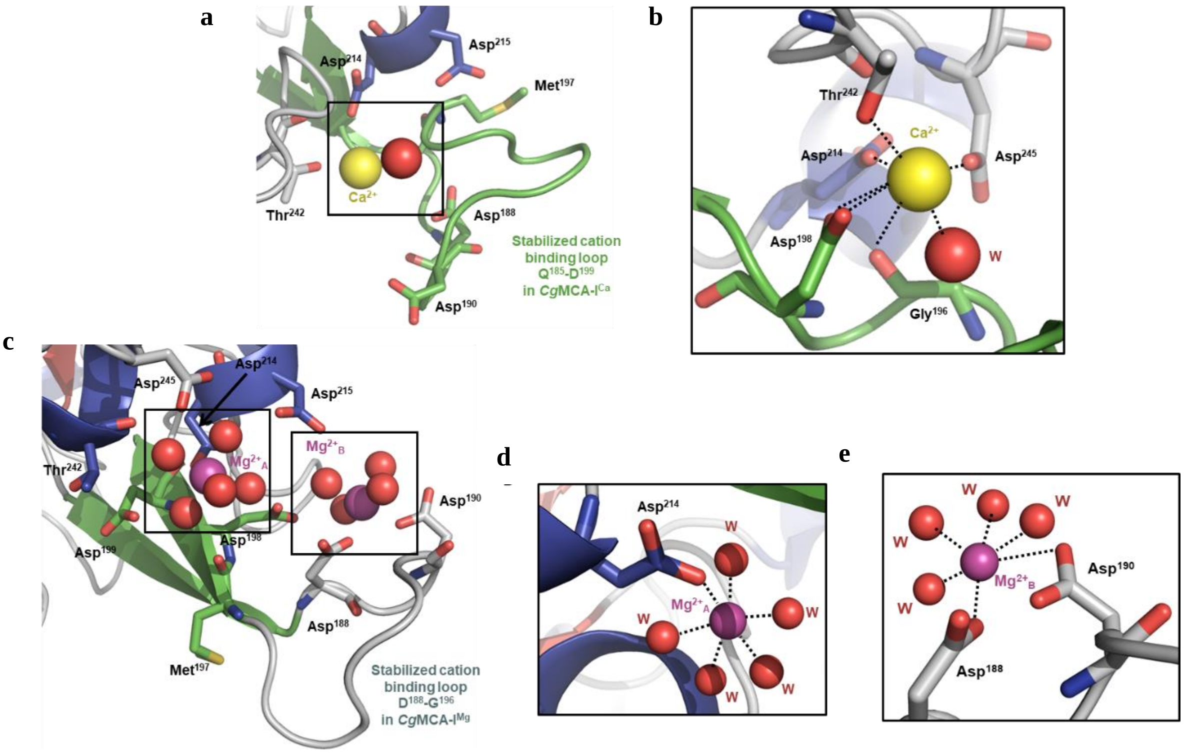

Supplementary Figure 10. Comparison of the organization of the cation binding loop as seen in *Cg*MCA-I^{Ca} and in *Cg*MCA-I^{Mg}. a. Overall organization of the *Cg*MCA-I^{Ca} cation binding loop. **b.** Ca^{2+} coordination involving residues side chains of Gly^{196}, Asp^{198} (2 coordinate bonds), Asp^{214}, Thr^{242}, Asp^{245} and a water molecule for a coordination number of 7. **c.** Overall organization of the *Cg*MCA-I^{Mg} cation binding loop. **d.** Mg$^{2+}_{A}$ coordination by five water molecules and the side chain of Asp^{214}. **e.** Mg$^{2+}_{B}$ coordination by four water molecules and side chains of Asp^{188} and Asp^{190}.

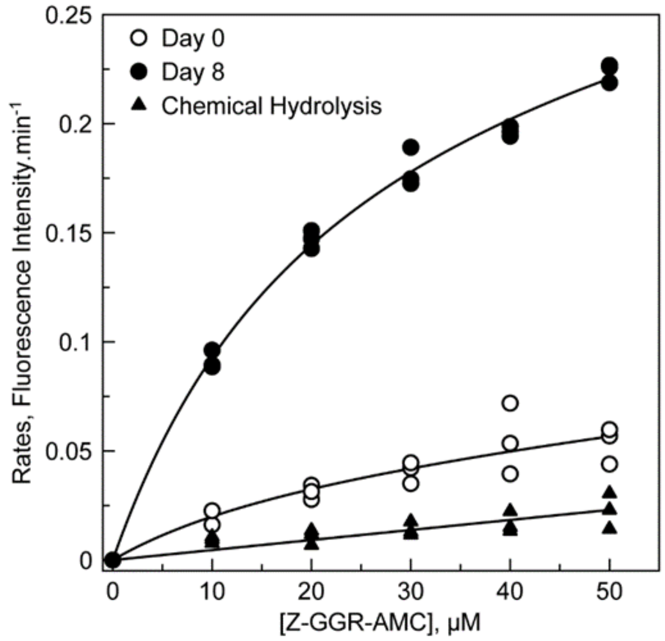

Supplementary Figure 11. Michaelis-Menten plot of non-matured (day 0 – shown as circles) and matured (day 8 – shown as bold circles) *Cg*MCA-I using Z-GGR-AMC as substrate (0-50 µM) without correction of the chemical hydrolysis (bold triangles). Reaction conditions: [*Cg*MCA-I] = 4 µM, [Z-GGR-AMC] = 0-50 µM, 10 mM HEPES buffer pH 7.6, 150 mM NaCl, 10 mM $CaCl_2$ (n=3).

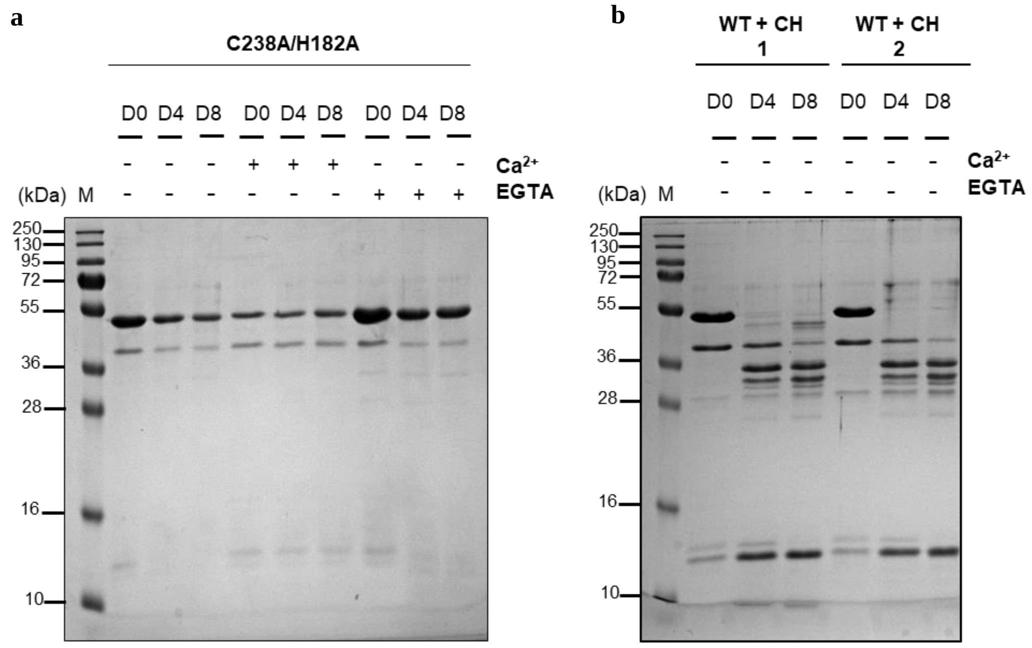

Supplementary Figure 12. Maturation of the double mutant C238A/H182A by the action of *Cg*MCA-I WT matured for 8 days. a. SDS-PAGE of aliquots of the double

mutant C238A/H182A at maturation day 0 (D0), 4 (D4) and 8 (D8) in the presence or absence of Ca^{2+} and EGTA. **b**. SDS-PAGE of aliquots of *Cg*MCA-I matured for 8 days with the double catalytic mutant C238A/H182A (CH) at D0, D4 and D8 of maturation in absence of Ca^{2+} and EGTA. Two ratios were used, a first mix of *Cg*MCA-I WT matured 8 days at 1 µM and 9 µM of CH (WT + CH 1) and a second mix of *Cg*MCA-I WT matured 8 days at 2 µM and 8 µM of CH (WT + CH 2).

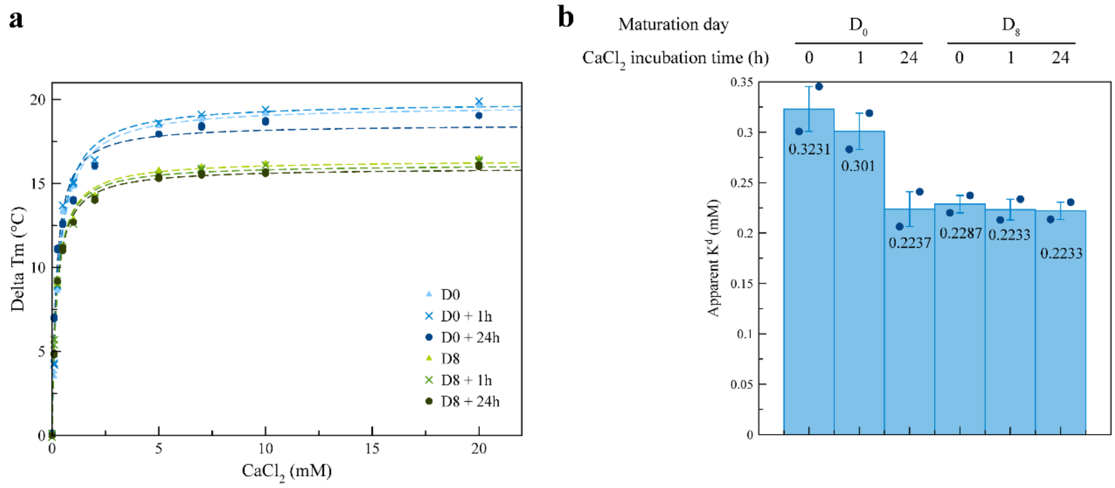

Supplementary Figure 13. Investigation of *Cg*MCA-I calcium affinity with different incubation time. a. Delta Tm variation of *Cg*MCA-I, at maturation day 0 and 8, in a range of $CaCl_2$ concentration without or with incubation time. Delta Tm are calculated from average Tm_0 (Tm without any ligand) for all three conditions, without and with 1 hour or 24 hours of incubation with different $CaCl_2$ concentrations priori to fluorescence measurements. All measurements were made in duplicate. **b.** Apparent K_d for *Cg*MCA-I at day 0 and 8 of maturation without or with 1 hour or 24 hours of incubation with $CaCl_2$. Apparent K_d were determined by curve-fitting using a single binding site model for calcium cations and are indicated with associated errors. Source data for **a** and **b** correspond to Supplementary Table 9 and Supplementary Table 10. **a.** symbols represent individual data points. **b.** blue dots represent individual data points and error bars represent standard deviation (SD) for n = 2 independent experiments. Mean values indicated on each bar.

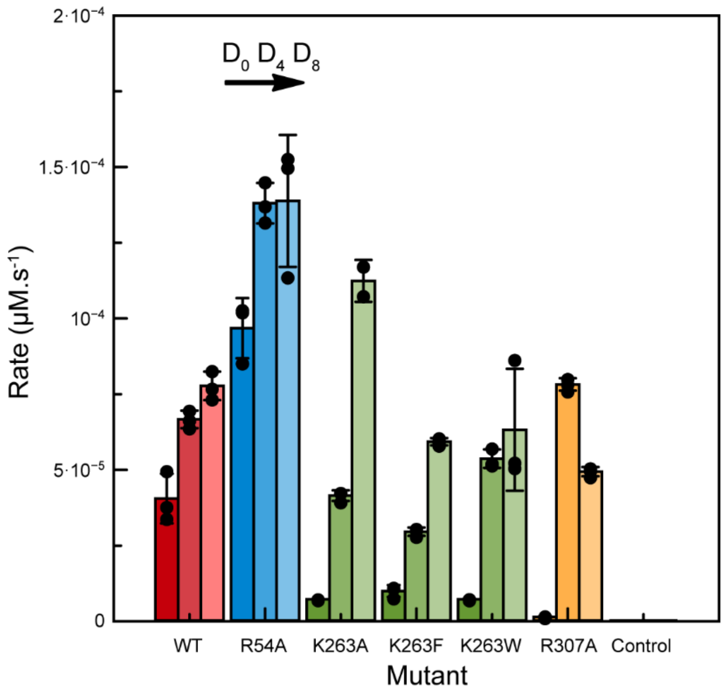

Supplementary Figure 14. Activity of wild type metacaspase and mutants determined at day 0 (D0), day 4 (D4) and day 8 (D8) of the maturation process. Reactions are performed in triplicate at 25°C in 10 mM HEPES buffer (pH 7.6, 150 mM NaCl, 10 mM $CaCl_2$) using 2 µM of enzyme and 50 µM Z-GRR-AMC. Control does not include enzyme. Black dots represent individual data points and error bars represent standard deviation (SD) for n = 3 independent experiments.

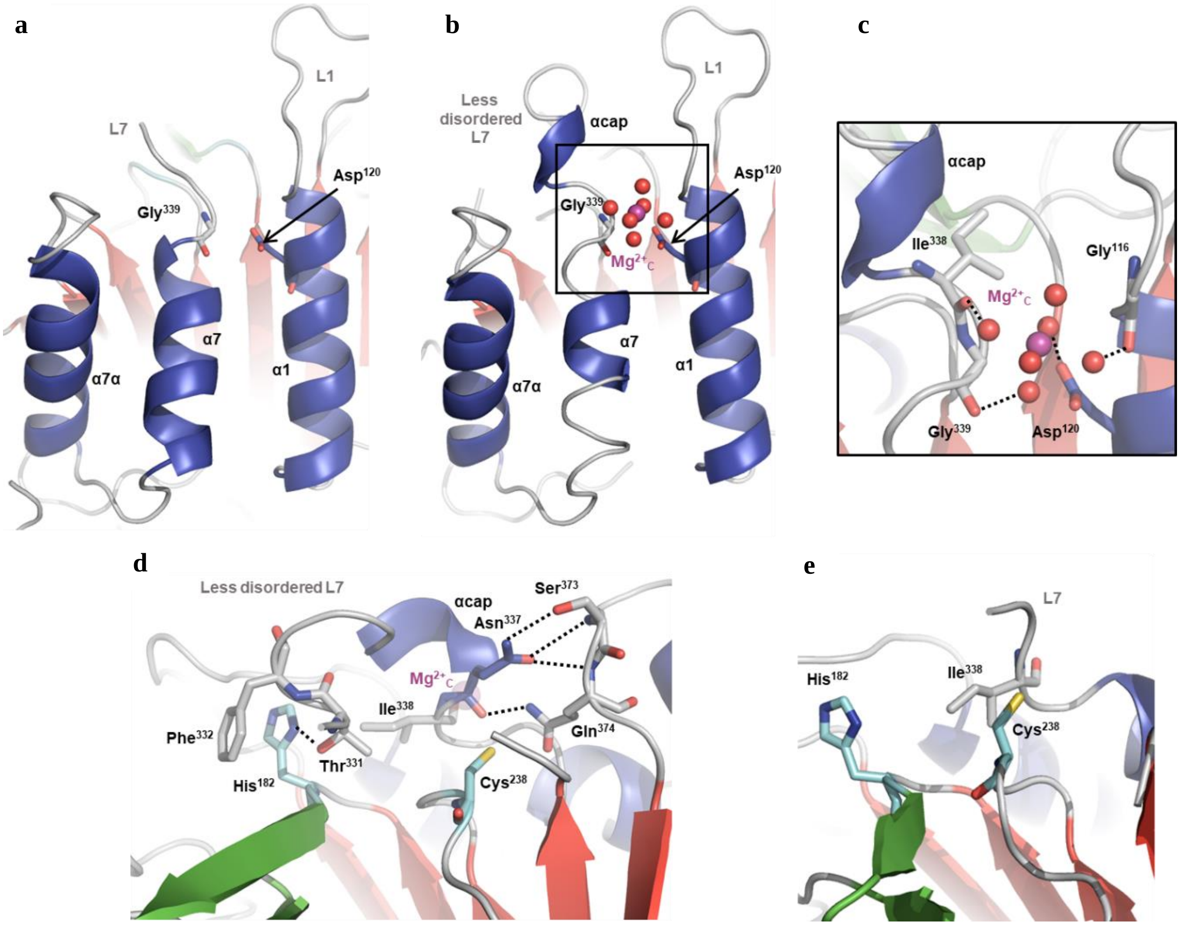

Supplementary Figure 15. L7 loop and α7 helix reorganization in *Cg*MCA-I^{Ca} and *Cg*MCA-I^{Mg}. **a**. Overall organization of *Cg*MCA-I^{Ca} L7 loop and α7 helix. **b**. Overall organization of *Cg*MCA-I^{Mg} L7 loop and α7 helix in the presence of $Mg^{2+}{}_C$. **c**. $Mg^{2+}{}_C$ coordination by six water molecules. Residues from α7 and α1 helices interacting with the water molecules are shown in stick (Main chain of Gly^{116}, Ile^{338} and Gly^{339} plus Asp^{120} side chain). **d, e**. L7 loop stabilization observed in *Cg*MCA-I^{Mg} with the formation of the αcap (**d**) compared to *Cg*MCA-I^{Ca} (**e**).

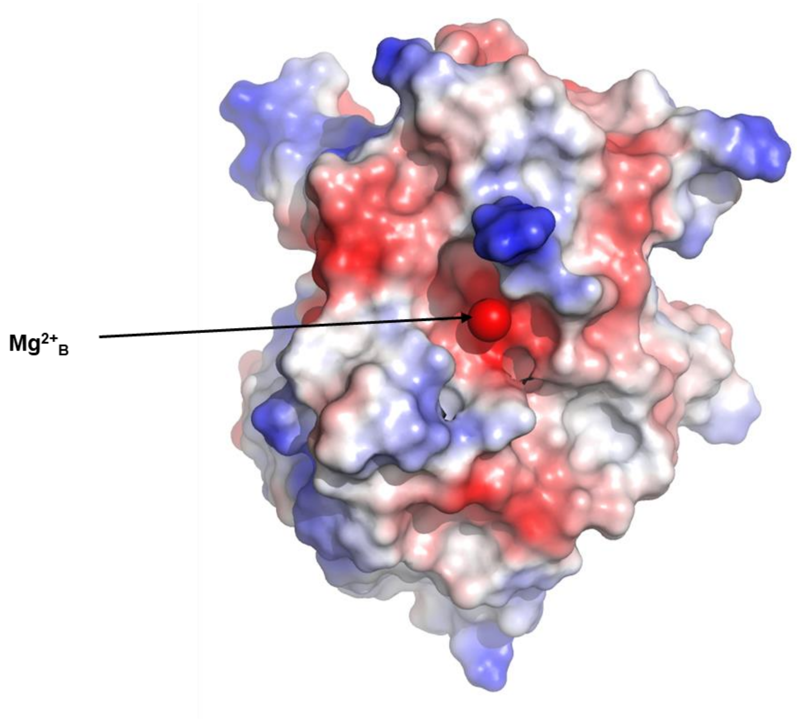

Supplementary Figure 16. Electrostatic surface potential of *Cg*MCA-I^{Mg}. The negatively and positively charged surface are respectively colored in *red* and *blue*. Mg^{2+} ion is shown as a *red* sphere.

Supplementary References

1. Sievers, F. *et al.* Fast, scalable generation of high-quality protein multiple sequence alignments using Clustal Omega. *Mol Syst Biol* **7**, 539 (2011).

2. Robert, X. & Gouet, P. Deciphering key features in protein structures with the new ENDscript server. *Nucleic Acids Research* **42**, W320–W324 (2014).

3. Wong, A. H.-H., Yan, C. & Shi, Y. Crystal Structure of the Yeast Metacaspase Yca1. *Journal of Biological Chemistry* **287**, 29251–29259 (2012).

4. McLuskey, K. *et al.* Crystal structure of a Trypanosoma brucei metacaspase. *PNAS* **109**, 7469–7474 (2012).
