# Supplementary material for: Structural and molecular determinants of Candida glabrata metacaspase maturation and activation by calcium
Source: Commun Biol. 2022 Oct 31;5:1158. doi: 10.1038/s42003-022-04091-4 (PMC9622860; doi:10.1038/s42003-022-04091-4)
Supplement: Supplementary file 3 — Description of additional supplementary files [file 42003_2022_4091_MOESM3_ESM.docx]

**Description of Additional Supplementary Files**

**File name**: Supplementary Data 1

Description: The source data for all enzymatic assays.

Source Data Fig 6

Source Data Sup Fig 11

Source Data Sup Fig 14

**File name**: Supplementary Data 2

Description: The uncropped and unedited gel images.

Source Data Fig 1

Source Data Fig 2

Source Data Sup Fig 1

Source Data Sup Fig 2

Source Data Sup Fig 4

Source Data Sup Fig 12
