# Supplementary material for: Structural and molecular determinants of Candida glabrata metacaspase maturation and activation by calcium
Source: Commun Biol. 2022 Oct 31;5:1158. doi: 10.1038/s42003-022-04091-4 (PMC9622860; doi:10.1038/s42003-022-04091-4)

Source Data Fig 1

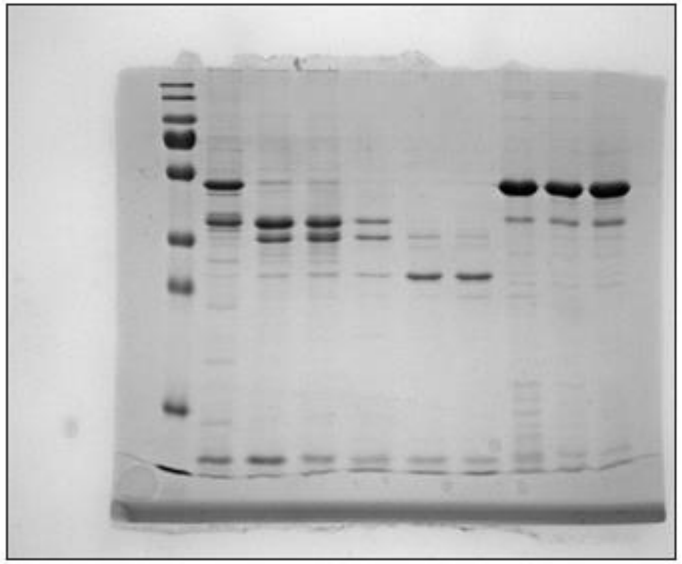

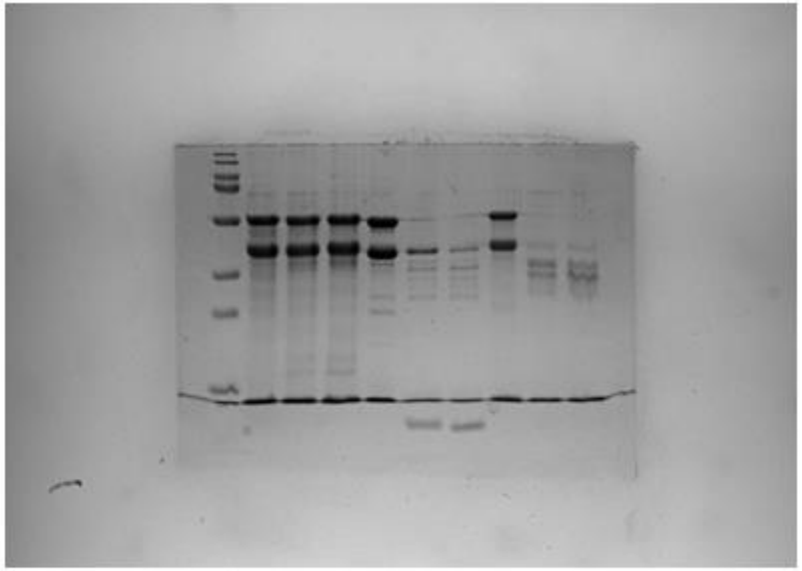

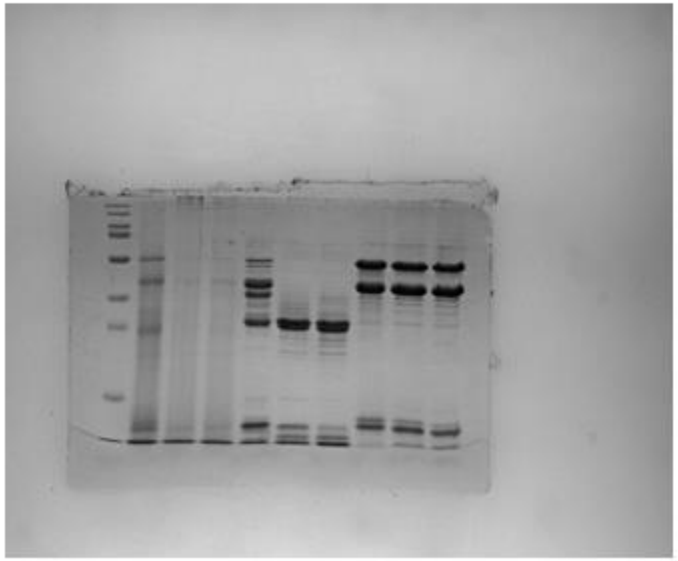

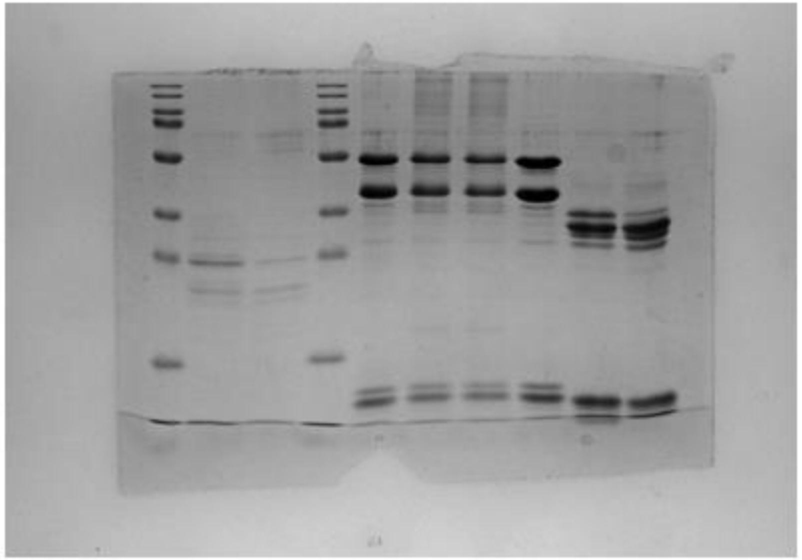

Source Data Fig 2

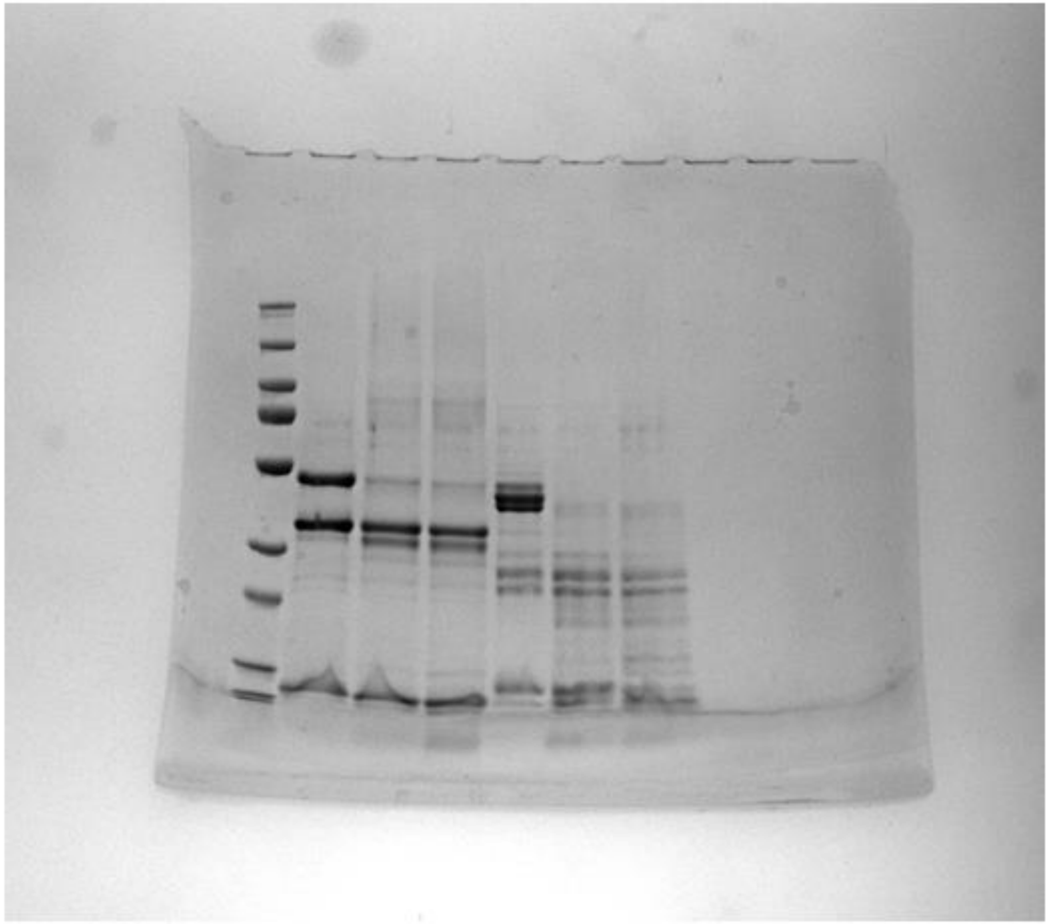

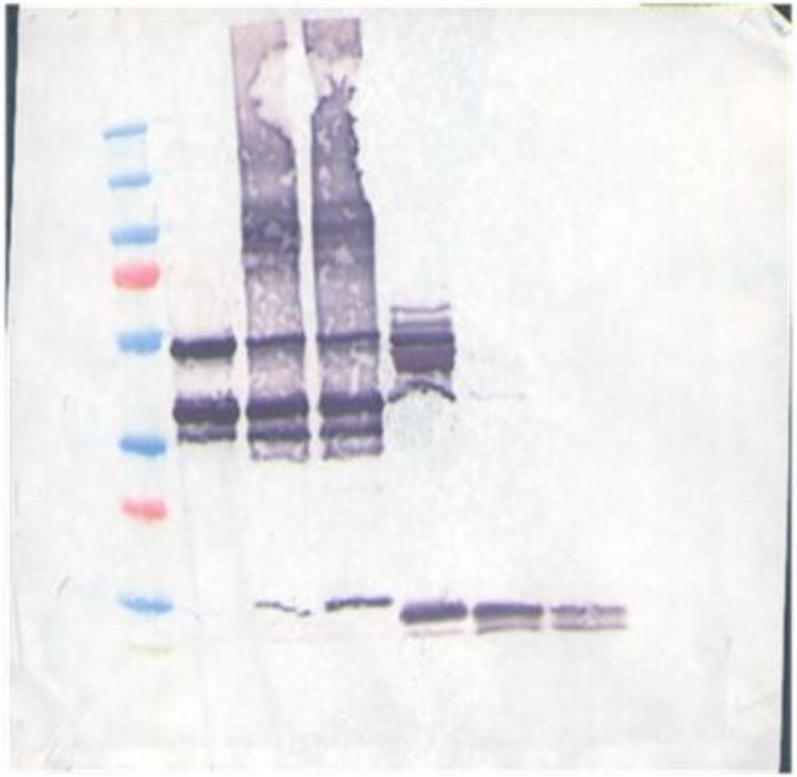

Source Data Sup Fig 1

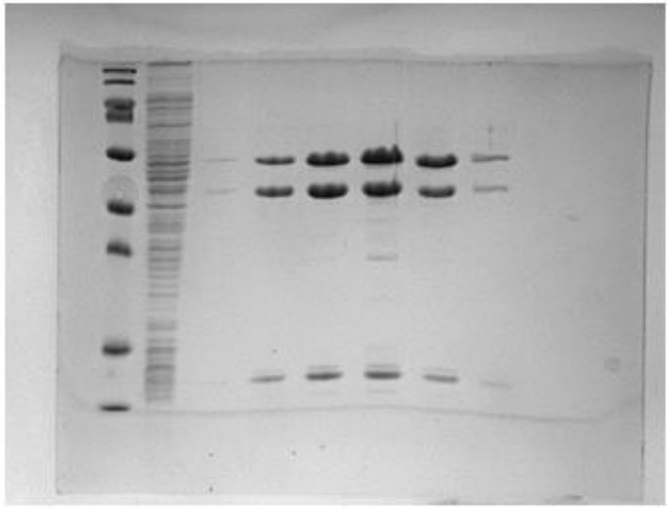

Source Data Sup Fig 2

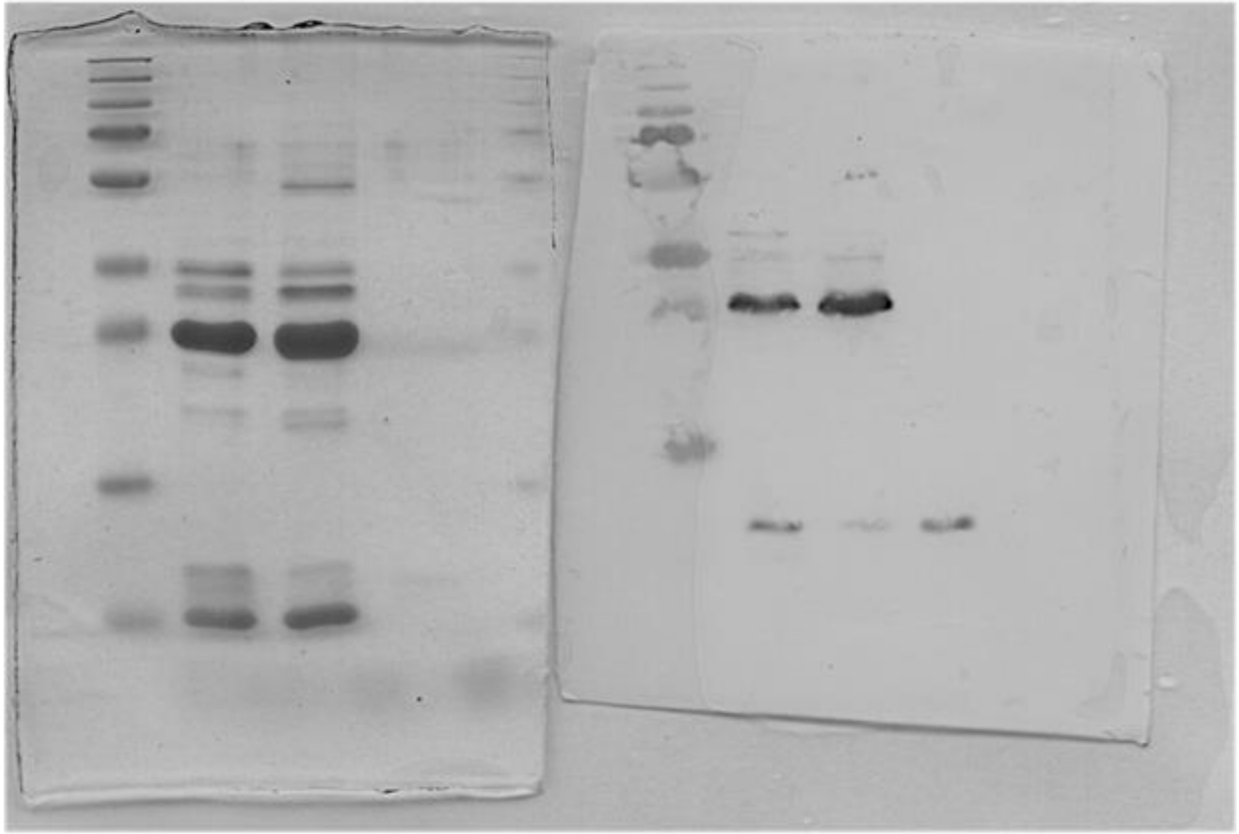

Source Data Sup Fig 4

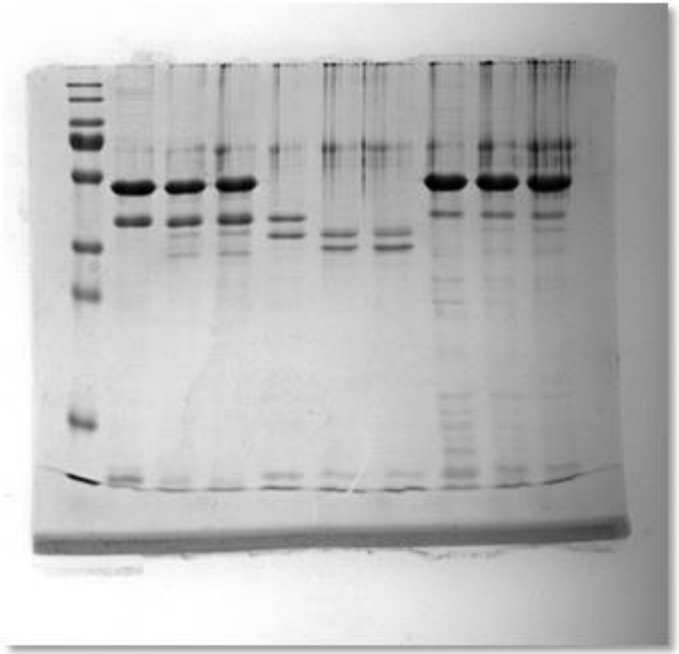

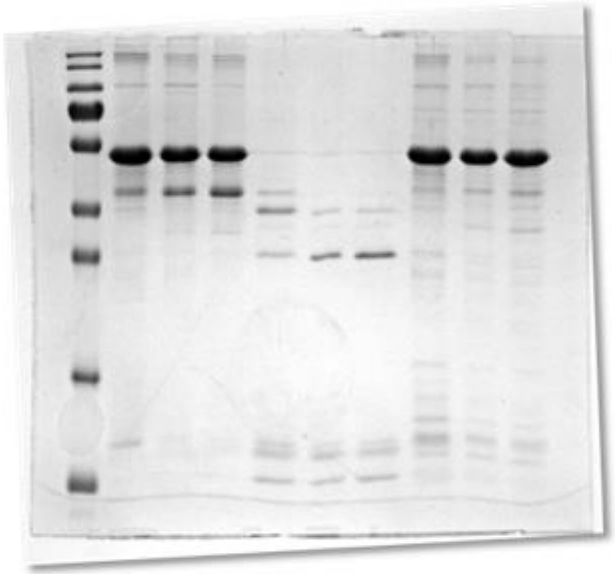

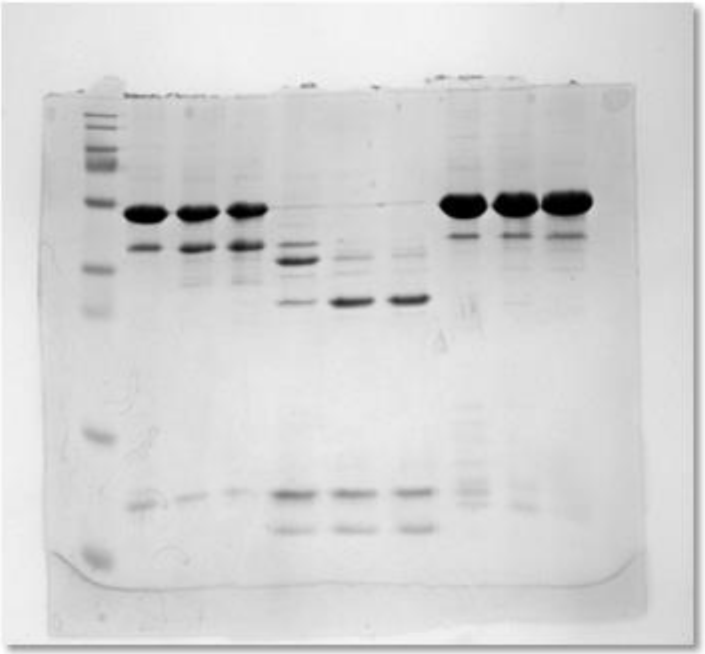

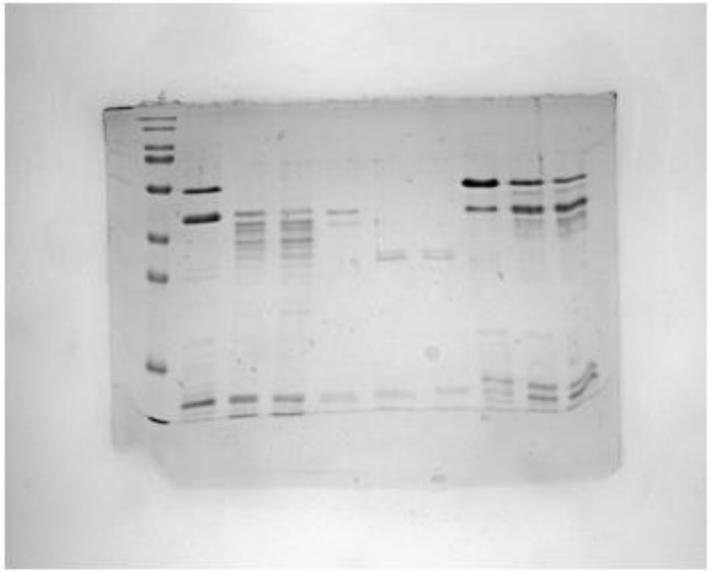

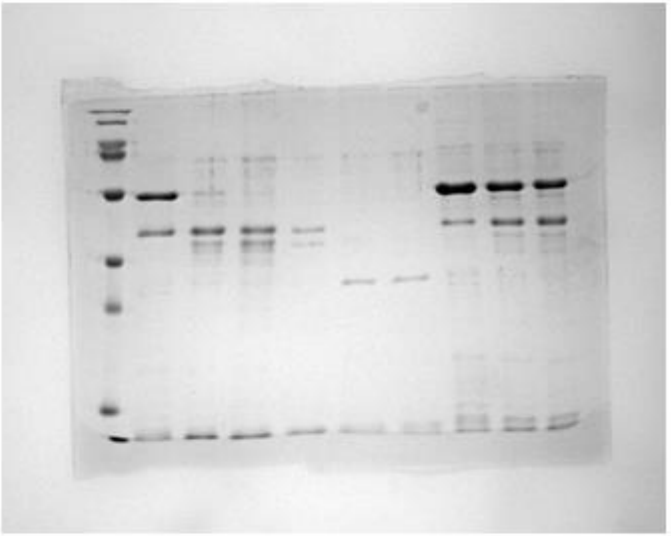

Source Data Sup Fig 12

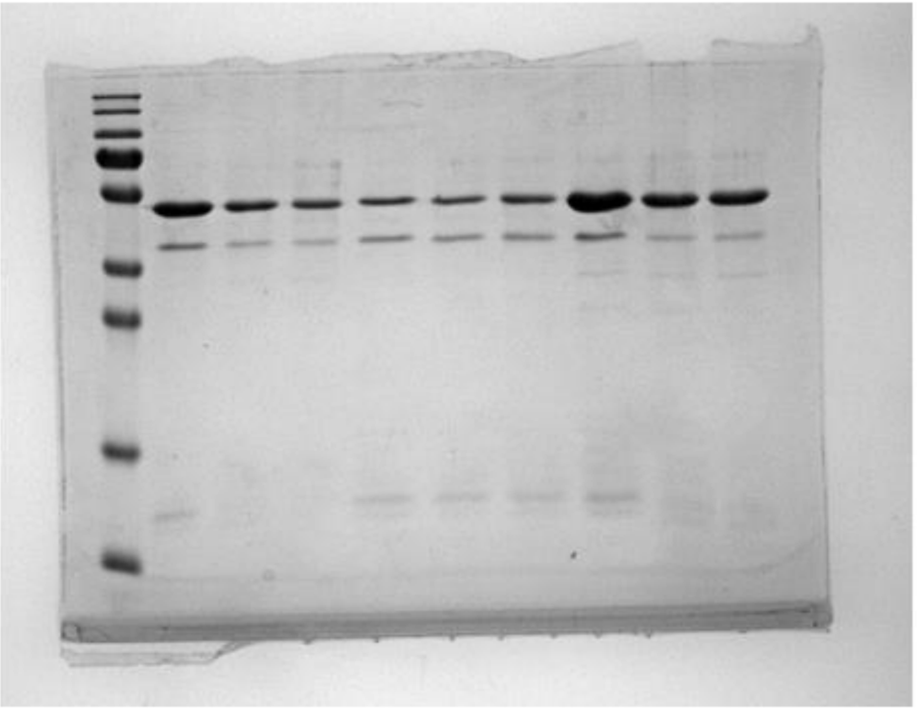

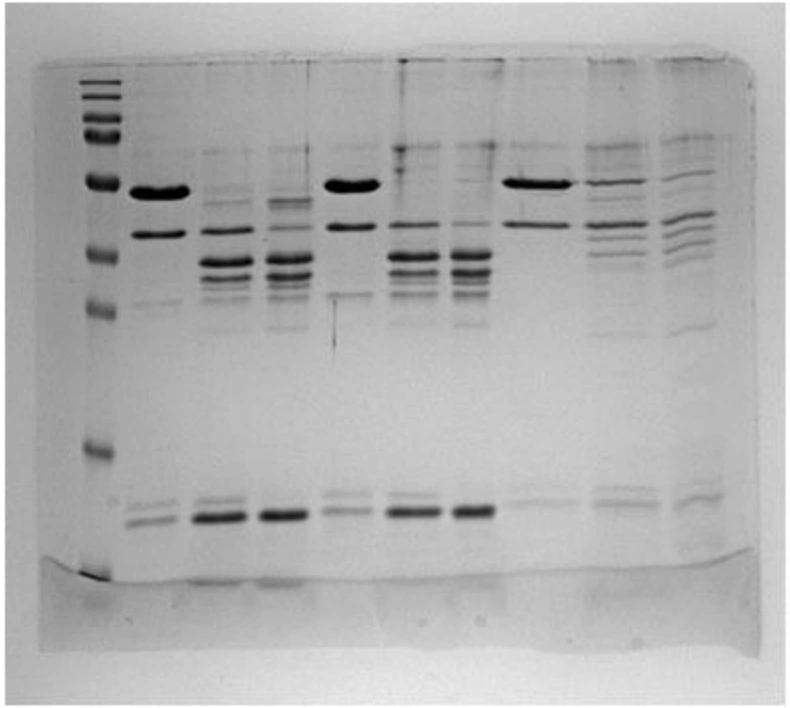

Supplement: Supplementary file 5 — Supplementary Data 2 [file 42003_2022_4091_MOESM5_ESM.pdf]
