# Supplementary material for: Structural and molecular determinants of Candida glabrata metacaspase maturation and activation by calcium
Source: Commun Biol. 2022 Oct 31;5:1158. doi: 10.1038/s42003-022-04091-4 (PMC9622860; doi:10.1038/s42003-022-04091-4)
Supplement: Supplementary file 6 — Reporting Summary-New [file 42003_2022_4091_MOESM6_ESM.pdf]

nature portfolio

Corresponding author(s): Lionel BALLUT, Nushin AGHAJARI

Last updated by author(s): Sep 26, 2022

Reporting Summary

Nature Portfolio wishes to improve the reproducibility of the work that we publish. This form provides structure for consistency and transparency in reporting. For further information on Nature Portfolio policies, see our Editorial Policies and the Editorial Policy Checklist.

Statistics

For all statistical analyses, confirm that the following items are present in the figure legend, table legend, main text, or Methods section.

n/a	Confirmed	
☐	☒	The exact sample size (*n*) for each experimental group/condition, given as a discrete number and unit of measurement
☐	☒	A statement on whether measurements were taken from distinct samples or whether the same sample was measured repeatedly
☒	☐	The statistical test(s) used AND whether they are one- or two-sided<br>*Only common tests should be described solely by name; describe more complex techniques in the Methods section.*
☒	☐	A description of all covariates tested
☒	☐	A description of any assumptions or corrections, such as tests of normality and adjustment for multiple comparisons
☐	☒	A full description of the statistical parameters including central tendency (e.g. means) or other basic estimates (e.g. regression coefficient) AND variation (e.g. standard deviation) or associated estimates of uncertainty (e.g. confidence intervals)
☒	☐	For null hypothesis testing, the test statistic (e.g. *F*, *t*, *r*) with confidence intervals, effect sizes, degrees of freedom and *P* value noted<br>*Give P values as exact values whenever suitable.*
☒	☐	For Bayesian analysis, information on the choice of priors and Markov chain Monte Carlo settings
☒	☐	For hierarchical and complex designs, identification of the appropriate level for tests and full reporting of outcomes
☒	☐	Estimates of effect sizes (e.g. Cohen's *d*, Pearson's *r*), indicating how they were calculated

Our web collection on statistics for biologists contains articles on many of the points above.

Software and code

Policy information about availability of computer code

Data collection	X-ray diffraction data were collected on the beamline PXIII (SLS) and PROXIMAI (SOLEIL). Enzymatic kinetics were recorded using a Tecan M200 microtiter plate reader (Salzburg, Austria) using black 96-well plates. SAXS data were collected at the SWING beamline (SOLEIL).
Data analysis	Data were indexed, integrated and scaled using XDS. The phase problem was solved by molecular replacement using phenix.phaser (PHENIX). The model was then built using Coot and refinement was carried out using PHENIX.<br>Regarding enzymatic assays, data analysis was performed and kinetic parameters were adjusted using the software package Dynafit.<br>SAXS data were processed using PRIMUS and calculation of P(r) was carried out using PRIMUS. Ab initio model envelopes of CgMCA-I were generated using DAMMIF. The envelopes were superimposed onto the crystal structure using the CHIMERA software (University of California, San Francisco).

For manuscripts utilizing custom algorithms or software that are central to the research but not yet described in published literature, software must be made available to editors and reviewers. We strongly encourage code deposition in a community repository (e.g. GitHub). See the Nature Portfolio guidelines for submitting code & software for further information.

Data

Policy information about availability of data

All manuscripts must include a data availability statement. This statement should provide the following information, where applicable:

- Accession codes, unique identifiers, or web links for publicly available datasets
- A description of any restrictions on data availability
- For clinical datasets or third party data, please ensure that the statement adheres to our policy

The datasets generated during and/or analysed during the current study are available in the PDB repository (https://www.rcsb.org/)

Field-specific reporting

Please select the one below that is the best fit for your research. If you are not sure, read the appropriate sections before making your selection.

☒ Life sciences ☐ Behavioural & social sciences ☐ Ecological, evolutionary & environmental sciences

For a reference copy of the document with all sections, see nature.com/documents/nr-reporting-summary-flat.pdf

Life sciences study design

All studies must disclose on these points even when the disclosure is negative.

Sample size	n/a
Data exclusions	n/a
Replication	n/a
Randomization	n/a
Blinding	n/a

Reporting for specific materials, systems and methods

We require information from authors about some types of materials, experimental systems and methods used in many studies. Here, indicate whether each material, system or method listed is relevant to your study. If you are not sure if a list item applies to your research, read the appropriate section before selecting a response.

Materials & experimental systems

n/a	Involved in the study	
☐	☒	Antibodies
☒	☐	Eukaryotic cell lines
☒	☐	Palaeontology and archaeology
☒	☐	Animals and other organisms
☒	☐	Human research participants
☒	☐	Clinical data
☒	☐	Dual use research of concern

Methods

n/a	Involved in the study	
☒	☐	ChIP-seq
☒	☐	Flow cytometry
☒	☐	MRI-based neuroimaging

Antibodies

Antibodies used	Monoclonal Anti-polyHistidine, Clone HIS-1, Product Number SIGMA H1029; Anti-Mouse IgG (whole molecule)–Alkaline Phosphatase antibody produced in goat, Product Number SIGMA: A3562
Validation	Monoclonal Anti-polyHistidine (mouse IgG2a isotype) is derived from the HIS-1 hybridoma produced by the fusion of mouse myeloma cells and splenocytes from a BALB/c mouse immunized with a recombinant His-tagged fusion protein. The isotype is determined by a double diffusion immunoassay using Mouse Monoclonal Antibody Isotyping Reagents, Catalog Number ISO2. Monoclonal anti-polyHistidine recognizes native as well as denatured-reduced forms of synthetic polyhistidine or polyhistidine-tagged fusion proteins.
